# The impacts of climate change on occupational health and work among outdoor workers: A scoping review

**DOI:** 10.1371/journal.pgph.0005888

**Published:** 2026-02-06

**Authors:** Behdin Nowrouzi-Kia, Charlene Choi, Raihana Premji, Aishvinigaa Sathananthan, Kishana Balakrishnar, Alexia Haritos, Bao-Zhu Stephanie Long, Maryna Mazur, Ali-Bani Fatemi

**Affiliations:** 1 Department of Occupational Science and Occupational Therapy, Temerty Faculty of Medicine, University of Toronto, Toronto, Canada; 2 Krembil Research Institute-University Health Network, Toronto, Canada; 3 Centre for Research in Occupational Safety & Health, Laurentian University, Sudbury, Canada; 4 Institute of Health Policy, Management and Evaluation, University of Toronto, Toronto, Canada; PLOS: Public Library of Science, UNITED STATES OF AMERICA

## Abstract

Climate change has a significant impact on human health and productivity at work. Environmental changes, including extreme temperatures and natural disasters, contribute to psychological pressures and physical impairments which affect quality of life and well-being. This scoping review examines the effect of climate change on human health in occupational settings. A systematic search of MEDLINE, CINAHL, Embase, and PsycINFO identified empirical studies that analyzed the impacts of climate change on human health in relation to work. Eligible studies included employed individuals aged 18–65, peer-reviewed studies published between 2000 and 2024, and evidence linking climate change to occupational health outcomes. Studies underwent title, abstract, and full-text screening. The scoping review, registered with the Open Science Framework, includes 62 studies. Three overarching themes emerged: (1) climate change and mental health; (2) climate change and physical health; and (3) climate change and work. Research has demonstrated the association of psychological distress with extreme weather events, occupational stress, and anxiety among outdoor workers. Physical health risks associated with higher temperatures include chronic dehydration, heat-related illnesses, and other injuries. Climate change also negatively impacts work productivity, leading to increased labour shortages and subsequent economic losses. Climate change has complex effects on the physical and mental health of workers, with significant implications for productivity and safety in the workplace. Despite growing evidence, targeted interventions remain limited. Future studies should examine the long-term health consequences, develop standardized alleviation strategies, and implement policies to protect employees from climate-related occupational hazards.

## Introduction

### Background

Climate change is a global phenomenon, referring to long-term changes to a region’s average weather patterns, including rising global temperatures, and changes in air quality (e.g., increased CO_2_ levels) and precipitation patterns [1,2]. These changes can lead to rising sea levels, increased storm severity, and prolonged droughts [3]. As a result, climate change exacerbates existing public health challenges by increasing exposure to new pathogens, deteriorating air and water quality, and amplifying health risks [3,4]. With the population growing rapidly, human activities, particularly industrialization and resource consumption, have significantly contributed to these environmental changes. According to Statistics Canada, households accounted for approximately 17.5% of Canada’s total greenhouse gas (GHG) emissions in 2020 [5].

GHC emissions are further increased by industrial sectors such as agriculture, manufacturing, energy production, and transportation, which disrupt atmospheric processes and accelerates climate change [6]. While some literature has focused on the physical challenges of climate change, a significant research gap remains regarding its effects on workers’ mental health and job performance. To bridge this gap, this review will examine physical, mental and work-related impacts as interconnected and equally important factors. This approach will support the development of effective interventions that mitigate occupational health risks.

### Climate change and mental health

Research increasingly highlights the strong association between mental health and climate change. Exposure to extreme weather conditions has been associated with adverse psychological outcomes. For instance, Chen et al. analyzed health records from Taiwan’s national longitudinal health insurance database [7]. Focusing on individuals without a prior diagnosis of major depressive disorder (MDD). Findings indicate that individuals living in regions with extreme temperatures (>23°C) experienced a 7% increase in MDD incidence per 1°C increment [7].

Similarly, Obradovich et al. examined the mental health effects of short-term exposure to extreme weather among two million U.S. residents (2002–2012) [8]. Their study found that higher monthly temperatures (>30°C) and natural disasters (e.g., wildfires, floods, hurricanes) significantly amplified psychological distress [8]. These studies demonstrate that the combined effects of increased environmental pressures, resulting from rising temperatures, create environmental stressors that pose a threat to human mental health [8]. Climate change has exacerbated this issue, necessitating a deeper investigation into its impact on human health and productivity.

### Climate change and physical health

Climate change presents major challenges to human health, particularly due to rising temperatures that increase the likelihood of heat-related illnesses (HRIs) [9]. These risks are augmented when the body’s temperature regulation is impaired, resulting in unfavourable conditions, including heat rash, cramps, severe heat stroke and exhaustion. Gibb et al. demonstrated the effect of heat on kidney health, showing that increased temperatures led to an increased risk of acute kidney injury [9]. Furthermore, increased heat also caused a decrease in renal function, potentially due to dehydration as well as worsening pre-existing illnesses and injuries [9]. Workers are particularly vulnerable to HRIs due to their occupational environments. Agricultural workers, for instance, face heightened risks of kidney damage due to heavy workloads in extreme heat [10]. Furthermore, research has also shown that death due to HRIs among construction workers is 13 times more likely, compared to workers in other industries; heat-related mortality among agricultural workers is 35 times higher [11,12]. These results demonstrate the critical impact climate change has on physical health.

### Climate change and work

Climate change has consequences that extend beyond health, impacting workplace behaviors and productivity. Research by Brooks and Greenberg suggests that extreme weather events can have significant psychological and occupational impacts. For example, there have been reports of increased job tension, higher turnover rates, and hostility in the workplace [13]. Furthermore, extreme weather-induced stress can impact decision-making and may result in greater workloads [13].

The intersection of mental health, work, and climate change has been scarcely examined in the literature [13]. Extreme climate and weather conditions play a crucial role in how quality of life and wellness is generally perceived. Concerns regarding climate crises often contribute to anxiety about the future and impact vulnerable populations by exacerbating existing health issues and amplifying socioeconomic stressors.

### Objectives

This review will examine the widespread effects of climate change on work and mental and physical health among outdoor workers. To the best of our knowledge, there are no existing knowledge syntheses that have investigated the impacts of climate change on human health and work, and none have proposed solutions to mitigate these impacts.

## Methods

### Study design

We conducted a scoping review to synthesize existing research on the challenges posed by climate change to human health. This scoping review was conducted by adhering to the Preferred Reporting Items for Systematic reviews and Meta-Analyses extension for Scoping Reviews (PRISMA-ScR) checklist (S1 Checklist) [14] and was registered with the Open Science Framework (DOI: https://doi.org/10.17605/OSF.IO/UM86E).

### Search strategy

Four databases—Medline, CINAHL, Embase (Ovid), and APA PsycINFO (Ovid)—were searched and updated on August 20^th^, 2025, for literature concerning climate change, human health, and work. The search terms were developed in collaboration with a Health and Society librarian from the University of Toronto (see Table 1). Two authors developed the search syntax (BNK, AH), while all authors participated in peer-review. See S1 Table through S4 Table for the complete search syntax in all four databases.

**Table 1:** Search syntax samples for the preliminary search.

Line #	Syntax
**1**	({Occupational Stress} OR {Job Stress} OR {Workplace Stress} OR {Job Fatigue} OR {Psychological Stress} OR {Mental Health} OR {mental disorder} OR {mental illness} OR {Physical Health} OR {Physical Fatigue} OR {Physical stress} OR {Emotional Exhaustion} OR {mental fatigue} OR {stress} OR {fatigue} OR {“quality of life, distress, trauma”} OR {Work Performance*} OR{Job Performance*} OR{Work engagement} OR {Job accommodation} OR {job satisfaction} OR {“work, health and safety”})
**2**	AND ({Climate Change} OR {Climate*} OR {Global Warming} OR {pollution} OR {air pollution} OR {Change, Climate} OR {Climate emergency} OR {Global Climate Change} OR {Climate Crises} OR {greenhouse gas*} OR {Increased gas emission} OR {Global Heating} OR {natural disaster*} OR {greenhouse effect})
**3**	AND ({Occupation*} OR {working women} OR {working men} OR {Work*} OR {workplace*})

### Inclusion and exclusion criteria

The eligibility criteria for this review were determined using the Population, Concept and Context (PCC) framework, consistent with guidance for scoping reviews. These criteria included the following types of studies: descriptive, cross-sectional, case-control, retrospective observational, quasi-experimental, experimental, longitudinal, cohort, time-series, ecological, randomized controlled, field, or quality improvement designs. Non-peer-reviewed articles, non-empirical articles, tertiary studies, dissertations, and pilot studies were excluded. This review focused specifically on outdoor workers aged 18–65, as this population is disproportionately exposed to climate-related occupational risks, including extreme heat and air pollution (see Table 2).

**Table 2:** Overview of the PCC Framework: Inclusion and Exclusion Criteria.

PCC Framework	Inclusion Criteria	Exclusion Criteria
Population	Adults aged 18–65, who are currently employed (full-time or part-time)	Adults under the age of 18, or 18+ but unemployed
Concept	Impact of climate change on physical health and mental health and in relation to occupational health and work	
Context	Studies published between the year 2000–2024	Studies published in the year <2000
	Full-Text publications in English-language	Non-English language publications

### Data collection

Using the Covidence software [15], a comprehensive literature review was independently conducted to identify pertinent studies based on established inclusion and exclusion criteria. The screening process included an initial review of the title and abstract, followed by a full-text review. Discussions among the reviewers were held to resolve discrepancies, with a consensus reached either through mutual agreement or by consulting with the senior author (BNK) and the research team. The selected articles were organized using the Zotero reference manager [16].

### Data extraction

Five reviewers (BNK, B-ZSL, AH, MM, and KB) independently extracted data from the included studies. Pilot testing was performed prior to the extraction to ensure inter-rater agreement among the reviewers and the style in which the findings were reported. Data were charted into Microsoft Excel, which included the following categories: 1) study title, 2) citation of the authors, 3) publication year, 4) country, 5) study objective(s), 6) study design/methodology, 7) total sample size, 8) population demographics, 9) measures/tools, 10) climate change impact on physical health, 11) climate change impact on mental health, and 12) climate change impact on work. The reviewers performed cross-checking after the extraction process to ensure consistent results. Any disagreements that arose during the extraction process were discussed and settled using consensus by the first author (BNK) in collaboration with the team.

### Data synthesis

The data extraction focused on evaluating information related to population demographics, methods, and important findings on the effects of climate change on outdoor work. Five independent reviewers (BNK, B-ZSL, AH, MM, and KB) collaborated to identify key themes for a thorough literature review. The performance of a descriptive thematic analysis yielded key themes based on the Braun and Clarke framework [17]. The researchers also methodically employed a six-step procedure to carefully encode and enhance the data into cohesive themes, ensuring validation of each theme against the coded extracts and complete data collection. The researchers conducted detailed discussions to support this procedure, further refining and finalizing the themes.

## Results

**Fig 1:**
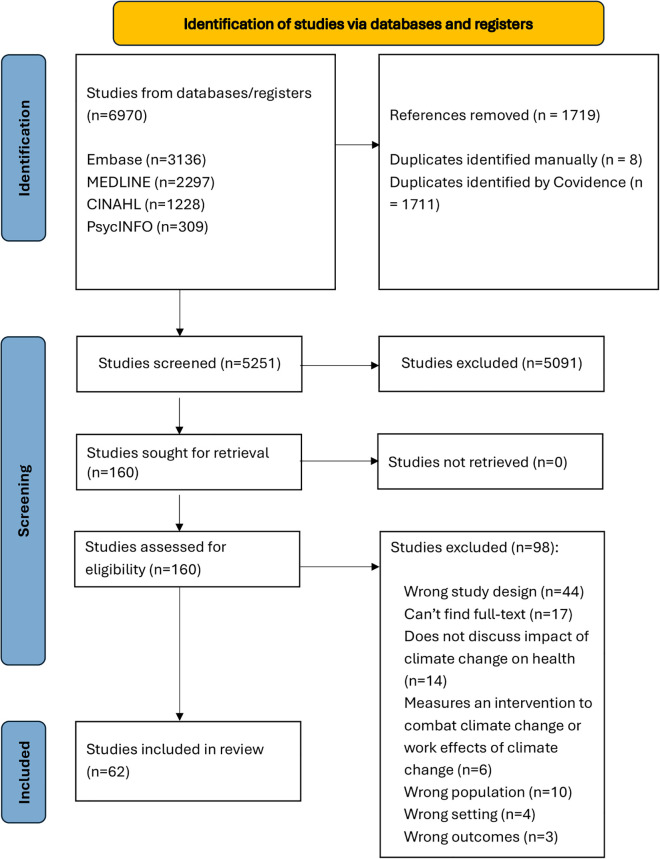
PRISMA Diagram.

In this scoping review, the titles and abstracts of 5,251 studies were screened. A total of 5,091 studies were then excluded as they did not fulfill our inclusion criteria, leaving 160 articles for full-text review. After completing the full-text review, sixty-two studies were identified for inclusion in our review. Fig 1 shows the process in detail.

### Study characteristics

A total of 62 studies were included in the analyses, which were conducted in 26 countries. These countries include, Canada, Saudi Arabia, Bahrain, the United Kingdom, Egypt, India, the United States, Germany, China and Costa Rica. In addition, Guatemala, Australia, Brazil, New Zealand, Southeast Asia, Vietnam, southern India, Taiwan, Nigeria, Ghana, Qatar, Nepal, Italy, Trinidad and Tobago, Ghana, Zambia, Mexico, Zimbabwe, and Thailand were also represented. Among these studies, 45 quantitative studies (including cross-sectional, cohort, time-series, and observational designs) provided a quantitative overview of various heat-related and climate-related factors influencing workers’ health conditions, 7 qualitative studies delved into personal experiences and risk factors of working under heat, 10 mixed-methods studies examined both broader statistical trends and personal narratives of working in heat conditions, and one was a quasi-experimental study offering insights into the effect of increased temperatures on workers’ heat stress. Detailed characteristics for all included studies can be found in Table 3.

**Table 3:** Summary of study characteristics of the studies that examine the impact of climate change on outdoor workers.

Author, Year	Country; Study Design	Study Population	Exposure(s); Quantification	Outcome(s)
Al-Bouwarthan et al., 2019	Saudi Arabia; Mixed-Methods Study	Construction workers	Heat exposure; WBGT	HRI
Al-Sayyad & Hamadeh, 2014	Bahrain; Cross-Sectional Study	Expatriate outdoor workers	CRC; Proportion of hospital visits and prescribed medications	HRI
Ayyappan et al., 2009	India; Case Study	Manufacturing workers (Outdoor locations)	Heat stress; WBGT	HRI; Stress; RP
Berman et al., 2021	United States; Cohort Study	Farmers	Drought conditions; SPI	Stress; RP
Brode et al., 2018	Germany; Simulation Study	Solar energy workers	Heat exposure; WBGT	Injuries; RP
Crowe et al., 2010	Costa Rica; Observational Study	Sugarcane workers	Heat stress; WBGT	RP
Dally et al., 2020	Guatemala; Quantitative Study	Agricultural workers	Heat exposure; WBGT	Injuries; RP
Gellert et al., 2022	Australia; Quasi-experimental intervention study	Construction workers	Heat stress; Non-exertional heart rate	HRI; Stress
Goodman et al., 2023	Australia; Mixed-Methods Study	Outdoor employees and contractors	Heat exposure; WBGT	Injuries; Fatigue; RP
Han et al., 2021	China; Cross-Sectional Study	Construction workers	Heat stress; Qualitative interviews	Injuries; RP
Hansen et al., 2020	Australia; Qualitative Study	Outdoor workers (i.e., construction, transport, manufacturing, agriculture and emergency services)	Heat exposure; Qualitative interviews	Injuries; Stress; RP
Hawkins & Ibrahim, 2023	United States; Quantitative Study	Outdoor workers (i.e., farming, fishing, forestry, construction)	Heat exposure; Data from NOAA	HRI and Injuries; RP
Hunt et al., 2023	Australia; Quantitative Study	Outdoor workers	Heat exposure; WBGT	RP
Ireland et al., 2023	Australia; Quantitative Study	Outdoor workers (i.e., agriculture, forestry, fishing, construction)	Heat exposure; Data from Bureau of Meteorology	Injuries
Kjellstrom et al., 2016	New Zealand; Quantitative Study	Outdoor workers	Heat exposure; WBGT	RP
Kjellstrom et al., 2013	South-East Asia; Modeling Study	Outdoor workers	Heat exposure; WBGT	HRI; RP
Krishnamurthy et al, 2017	Southern India; Observational study (quantitative and qualitative data collection)	Steel workers (Outdoor locations)	Heat stress; WBGT, dehydration levels	HRI and Fatigue; Stress; RP
Le Dang et al., 2014	Mekong Delta, Vietnam; Structured interviews	Farmers	Perceived risk of rising sea levels; Qualitative interviews	Injuries; Anxiety, Depression and related symptoms and Stress; RP
Lee et al., 2018	South Korea; quantitative analysis	Outdoor laborers	Heat stress; WBGT	HRI; RP
Lin & Chan, 2009	Taiwan; retrospective observational design	Outdoor workers (i.e., construction workers, farmers, fishers)	Heat exposure; Historical meteorological data	HRI; RP
Lohrey et al., 2021	Vietnam; Quantitative Study	Outdoor workers (i.e., construction workers, street vendors, shippers)	Heat exposure; KAP survey	HRI; Stress; RP
Lundgren et al., 2014	Chennai India; Case study	Industrial and agricultural workers	Heat stress; WBGT	HRI; Anxiety, Depression and related symptoms and Stress; RP
Lundgren-Kownacki et al., 2018	India; Mixed Methods	Brick kilns workers	Heat stress; WBGT	HRI; RP
Mansor et al., 2019	Negeri Sembilan; Cross Sectional Design	Outdoor workers (i.e., sweeping, cleaning drainage, cutting grass)	Heat exposure; WBGT	HRI; Stress; RP
Marinaccio et al., 2019	Italy; Epidemiological study	Agricultural and other outdoor workers (i.e., construction, fishing, mining)	Heat exposure; Data from NASA Terra satellite, Ta, monitoring networks and spatio-temporal land use	Injuries; RP
Mathee et al., 2010	South African; Qualitative Study	Outdoor workers (i.e., grave diggers, street sweepers, construction workers)	Heat exposure; Qualitative interviews	HRI; Stress; RP
McInnes et al., 2017	Australia; time-stratified case-crossover study	Outdoor workers	Heat exposure; Historical meteorological data	Injuries
Meade et al., 2017	Canada; case report	Male electrical utilities workers (Outdoor workplace)	Heat exposure; WBGT	HRI, Injuries and Fatigue; Stress; RP
Moore et al., 2025	Trinidad and Tobago; Qualitative intrinsic case study	Farmers	Heat exposure; Qualitative interviews	Anxiety, Depression and related symptoms and Stress
Mutic et al., 2017	USA (Florida); prospective cohort study	Farmworkers	Heat exposure; Data from FAWN	HRI; RP
Nag et al., 2013	India; observational field study design	Outdoor workers (i.e., stone quarry works)	Heat stress; WBGT	HRI
Nunfam et al., 2020	Ghana; Mixed-methods (focus discussion groups and questionnaire)	Mining workers	Heat stress; Qualitative interviews	HRI
Nunfam et al., 2019	Ghana; Mixed-methods (Questionnaire and focus groups)	Mining workers	Perceived heat exposure; Interviews and survey	HRI; Injuries and Fatigue
Nyambe, 2024	Zambia; Exploratory cross-sectional assessment (survey, group discussion and interviews)	Rural farmers	Perceived heat exposure and heat stress; Qualitative interviews	HRI; RP; Stress
Oyekale, A.S., 2015	Nigeria; Quantitative Study	Cocoa farmers	Climate-induced occupational stress; Survey	RP
Parker et al., 2024	USA; Qualitative Study	Agricultural workers	Heat exposure; Qualitative interviews	HRI; RP
Pires Biterncourt et al., 2020	Brazil; quantitative study	Outdoor workers	Heat exposure; Meteorological data, WBGT	HRI
Pogacar et al., 2019	Slovenia and Greece; cross-sectional study with longitudinal analysis	Outdoor workers (i.e., agriculture, construction, tourism)	Heat waves; Heat Wave Climatology	HRI; RP
Polain et al., 2011	Australia; qualitative study	Farmers	Perception of climate change and drought; Public forums	HRI
Pradhan et al., 2019	Qatar; quantitative study	Construction workers	Heat exposure; Data from CDHESD	HRI and Injuries
Pradhan et al., 2013	Nepal; observation survey	Agricultural workers	Heat stress; WBGT	HRI
Quiller et al., 2017	USA; cross-sectional study	Tree-fruit harvesters	Heat exposure; WBGT	HRI; RP
Rahimi et al., 2024	Iran; mixed methods	Outdoor workers (i.e., mine workers, construction, agricultural workers)	Heat exposure; Data from IMO	HRI and Injuries
Rahman et al., 2016	Bangladesh; cross-sectional	Construction sector and garments factory	Heat exposure; WBGT	HRI
Raval et al., 2018	Gujarat; mixed methods	Traffic police workers	Heat exposure; WBGT	HRI and Injuries; RP
Rudner et al., 2025	United States; longitudinal	Agricultural Workers	Survey; USG	HRI
Sahu et al., 2013	India; cross-sectional	Rice harvesters	Heat exposure; WBGT	HRI and Injuries
Sett & Sahu, 2014	India; cross-sectional	Female brick workers	Heat exposure; WBGT	HRI and Injuries
Samaniego-Rascon et al, 2019	Mexico; quantitative study	Solar workers	Heat stress; WBGT	Injuries
Schifano et al., 2019	Italy; association study	Outdoor workers (i.e., construction, transportation and energy workers)	Heat exposure and air pollution; Data from previous studies and REPA	Injuries
Shanmugam et al., 2023	Southern India; Cross-sectional study	Outdoor female workers	Heat exposure; WBGT, HIS	HRI
Spencer et al., 2022	Gambia; In-depth semi-structured interviews	Female pregnant farmers	Heat stress; Qualitative interviews	HRI and Injuries
Stoecklin-Marois et al., 2013	USA; Interviews	Farm workers	Heat exposure; Qualitative interviews	HRI
Sverdlik et al., 2024	Zimbabwe and India; Mixed Methods	Outdoor informal workers (i.e., waste pickers, vendors, urban farmers)	Extreme weather; Qualitative interviews	HRI; RP
Tawatsupa et al., 2010	Thailand; cohort study	Outdoor physical labour workers	Heat stress; Survey	HRI and Injuries; Anxiety, Depression and related symptoms
Varghese et al., 2019	Australia; time-stratified case-crossover	Outdoor workers (i.e., agriculture, fishing, farming, construction)	Heat waves; Meteorological data, EHF	HRI and Injuries
Venugopal et al., 2021	Southern India; cross-sectional study	Outdoor workers (i.e., agriculture, construction, brick)	Heat stress; WBGT, CBT, SwR, USG	HRI and Injuries
Venugopal et al., 2015	India; Mixed Methods	Outdoor workers (i.e., metal fabrication, agriculture, construction, brick)	Heat exposure; WBGT	HRI; RP
Wagoner et al., 2020	Mexico; cross-sectional study	Agriculture workers	Heat stress; WBGT, Ingested core body sensors, USG	Injuries; RP
Xiang et al., 2014	Australia; quantitative study	Outdoor workers (i.e., agriculture, forestry, fishing, mining, construction)	Heat waves; Meteorological data	Injuries
Xiang et al., 2016	Australia; cross-sectional study	Outdoor workers (i.e., agriculture, forestry, fishing, mining, construction)	Heat exposure; Survey	HRI and Injuries
Xiang et al., 2015	Australia; case-crossover	Outdoor workers (i.e., agriculture, forestry, fishing, mining, construction)	Heat waves; Meteorological data	HRI

**Abbreviations:** CBT: Core Body Temperature, CDHESD: Climate Data and Heat Exposure Software and Database, CRC: Climate Related Condition, including heat-related, allergic, infectious, gastroenteritis, other, eGFR: Glomerular Filtration Rate, EHF: Excess Heat Factor, FAWN: Florida Automated Weather Network, HIS: Heat Strain Indicator, HRI: Heat-Related Illness, IMO: Iran Meteorological Organization, KAP: Knowledge, Attitudes and Practices, NOAA: National Oceanic and Atmospheric Administration, REPA: Regional Environmental Protection Agency, RP: Reduced Productivity, SPI: Standardized Precipitation Index, SwR: Sweat Rate, Ta: Air Temperature, USG: Urine Specific Gravity, WBGT: Wet Bulb Globe Temperature

### Overarching themes

**Fig 2:**
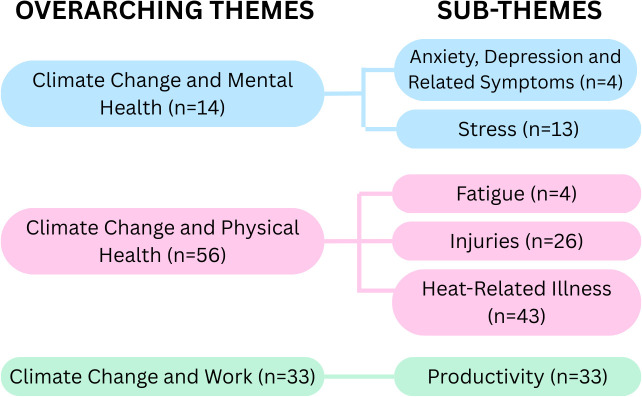
Concept map of identified themes from the impact of climate change on human health and work.

Of the 62 studies that were included in this review, 14 discussed mental health outcomes, 56 discussed physical health outcomes, and 33 discussed work-related outcomes. Refer to Fig 2 for a visual representation of the specific themes and sub-themes and their prevalence across the included studies. 29 of the included studies explored more than one theme. See S5 Table for a detailed breakdown of article distribution by major themes.

### Climate change and mental health

#### Anxiety, depression and related symptoms.

Four studies recognized the effects of climate change on outdoor workers’ anxiety, depression and related symptoms [18–20]. Moore et al. found that farmers generally reported negative feelings, including feelings of sadness, worry, discouragement, fear, frustration, hopelessness, and depression, due to fluctuating temperatures [18]. Moreover, an emerging trend seems to be that anxiety about climate change is interrelated with other personal, occupational, and geographic factors. Prolonged exposure to high-heat environments owing to workers’ geographic regions or the nature of their occupations can cause anxiety in workers [21,22]. Occupational factors have also been shown to influence anxiety, where outdoor workers may feel pressure from their superiors to maintain productivity regardless of weather conditions and related physical discomfort [21]. Furthermore, male workers have reported higher psychological distress, pointing towards gender as a significant covariate in the relationship between human health and climate change [20].

#### Stress.

Thirteen studies recognized the effect of climate change on stress in outdoor workers [18,19,23–33]. In fact, two studies reported that farmers perceived stress when working in hot conditions and experiencing changes in weather [18,23]. Berman et al. also found drought conditions are strongly associated with increased psychological stress among U.S. farmers, demonstrating the extent of interconnection between climate change and mental health [25]. In addition, personal beliefs and worries related to the outcome of climate change, including the loss of loved ones, personal property and belongings, financial losses, and production threats, are associated with increased mental health challenges [22]. In addition to personal challenges, climate change has also been shown to be stressful because of the significant toll that heat takes on the physical body. Research by Lohrey et al. highlighted how the physical strain of heat caused severe symptoms of heat stress, such as fainting and heat stroke [29]. Potential health risks and occupational challenges can be exacerbated by excess exposure to these conditions, ultimately contributing to augmented mental stress [26,29,32].

Climate-induced stress has also been shown to be heavily influenced by interactions between workers and supervisors. For instance, Hansen et al. found that superiors tend to put pressure on workers to maintain productivity levels regardless of weather conditions. Not only can this be physically harmful, but it also puts immense psychological stress on workers due to the severe health risks associated with long-term heat exposure [27]. These conditions can be especially damaging for younger workers who have difficulty asserting themselves to ensure their safety, resulting in reduced confidence and autonomy. These dynamics can be significantly burdensome, further highlighting the strong association between climate change, human health and work. Research has also shown that workplaces may lack sufficient strategies and protocols to navigate the harmful effects of heat exposure. These oversights, including limited access to air conditioning, can create greater barriers to worker mental health and productivity [29]. Other studies have offered recommendations for addressing these challenges and improving working conditions, encouraging employers to emphasize nutrition, self-pacing and more frequent breaks [26,28].

### Climate change and physical health

#### Fatigue.

Four studies recognized climate change’s effects on fatigue among outdoor workers [28,33–35]. Common concerns regarding heat-induced fatigue include productivity and reduced efficiency. The heat can take a severe physical toll on the body, often causing adverse and potentially dangerous effects due to fatigue and exertion, including shaky limbs, increased heart rate, headaches, and dehydration [21,34]. These symptoms are likely to limit productivity, with workers reporting difficulty focusing, more errors, longer task completion times, and general impaired cognitive functioning [28,33,34]. Research by Meade et al. showed that despite working at a slower pace due to heat, exertion was still rated at their normal level, illustrating how heat can increase fatigue among outdoor workers [33]. Fatigue due to heat exposure can also affect workers’ personal lives. For example, research by Krishnamurthy et al. showed that extended heat exposure can negatively impact workers’ social lives; some reported feeling too tired to socialize or dedicate time with family and friends [28].

#### Injuries.

Multiple studies have established an association between occupational injuries and high-temperature exposure [19,20,27,28, 33–54]. Heat exposure has been shown to weaken workers’ cognitive and motor functions, leading to higher risks of workplace accidents and injuries [34,37]. One study highlighted that agricultural workers faced a 3% increase in recorded injury risk for each increased degree in Wet Bulb Globe Temperature (WBGT) above 30°C, emphasizing the vulnerability of outdoor laborers in high-heat environments [37]. This study further highlighted the existence of a nonlinear relationship between WBGT and the risk of injury, where the risk of injury increases beyond certain temperature thresholds [37]. Additionally, research by Goodman et al. indicated that outdoor workers in industries including manufacturing and construction are more likely to sustain injuries due to heat-related strain and fatigue, supporting the need for improved workplace safety measures [34]. As global temperatures continue to rise, the correlation between climate change and occupational injuries emphasizes the urgency of implementing heat-mitigation strategies to protect worker health and safety [34,36,37].

#### Heat-related illnesses.

Heat-related illnesses are a significant concern in a multitude of occupational settings, especially in areas that experience high ambient temperatures. A study of Indian automotive workers highlighted that over 28% are at risk of heat-related health problems, especially during hotter periods, with temperatures as high as 42°C [24]. Additionally, Rudner et al. reported that 11% of workers experienced severe HRI symptoms after their shift during the summertime [55]. These findings align with those of Al-Bouwarthan et al., who have demonstrated that construction workers in Saudi Arabia are frequently exposed to excessive heat stress, resulting in increased visits to the emergency department for heat-related illnesses [56]. The study reported that conforming to midday work bans did not effectively reduce heat stress risks, indicating consistent vulnerability among outdoor workers [56]. Furthermore, Venugopal et al. revealed that the majority of salt-pan, construction, and brick workers experienced heat stress symptoms, such as excessive sweating, exhaustion, and dehydration [57]. Similar symptoms were also found in rural farmers when exposed to extreme heat, along with headaches, concentration loss, muscle cramps, hypertension, weakness, hunger, rashes, coughing and nose bleeds [23]. Some farmers believed their symptoms were caused by heat stress and heat-related illnesses [23]. Moreover, Nunfam et al., found that excessive sweating, headaches, heat exhaustion, heat rash and collapsing were all heat-related illnesses experienced by mining workers [58].

Gellert et al. similarly found that physiological stress significantly correlated with elevated ambient temperatures among outdoor workers, particularly in those with a higher body mass index (BMI) [26]. These findings collectively emphasize that, due to climate change amplifying these risks, there is an urgent need for effective heat stress management strategies across various industries. By establishing baseline data and implementing targeted interventions, employers can better protect their workforce from heat-related illnesses, improving overall occupational health and productivity [24,26,56].

### Climate change and work

#### Productivity.

Thirty-three studies have recognized the effects of climate change on workers’ productivity [19,23–25,27,29–34,36–39,41,45,59–74]. According to these studies, increased heat exposure is associated with decreased productivity among workers worldwide. For instance, in Southeast Asia, as much as 15% to 20% of working hours have been lost due to heat exposure, resulting in a loss of several percentage points of GDP and a reduction in labour productivity of up to 70% [61,75]. Nyambe also found that farmers perceived heat exposure and heat-related illness as negatively impacting their productivity [23]. Additionally, Bröde et al. conducted a study that revealed that heat exposure was too severe at certain points of the day, which required a loss of up to two hours per workday [36]. Furthermore, labor productivity is projected to decrease in the coming years. Research by Lee et al. showed that labor productivity was projected to decline by 26.1% in South Korea as temperatures continue to rise [64].

Current safeguards against health risks and barriers to productivity are insufficient for protecting the mental and physical health of outdoor workers, with many workplaces and employers being underequipped for managing high temperatures [65]. In fact, these studies reveal that many employers prioritize productivity over safety, often using intimidation and monetary incentives, which can be particularly harmful to vulnerable workers due to economic challenges and their citizenship status [70,74,76]. This excess pressure and toll on workers tend to exacerbate productivity shortages, as workers turn to absenteeism and sick leave, experience wage losses, require more breaks, work at a slower pace, manage health challenges, and undergo overall adjustments to their work habits [24,30,38,45,52,77,78].

## Discussion

Our scoping review examines the impact of climate change on human health and work. There is little research regarding the effects of climate change on a person’s mental health and well-being, particularly in terms of occupational health. This review will present the literature to demonstrate what we currently know, highlight gaps, and propose strategies to improve occupational health. Three key themes regarding the impact of climate change on human health and work were identified. These themes included *climate change, mental health,* *physical health,* *and work*.

### Climate change and mental health

Investigating the association between climate change and mental health, our results highlight various interrelated factors that have significant implications for the mental health of outdoor workers. Factors such as geographic location, personal concerns about the consequences of climate change, worries about physical and mental health risks, and top-down pressure to maintain productivity collectively contribute to workers’ worsening mental state amid climate change [21,22,26,27,29,32]. Schwartz et al. found that eco-anxiety and generalized anxiety disorder were significantly associated with each other, with reports of anxiety regarding climate change-related disasters and how their functioning (e.g., participation in activities of daily living and instrumental activities of daily living) seemed pointless in consequence of the negative impacts of climate change [79]. These findings support our previous finding that geographic location and existing beliefs about climate change can lead to reduced job engagement and exacerbate existing anxiety.

Ahmed et al. also found that many residents of coastal Bangladesh reported significant losses of land and loved ones, discussing how their severe financial and emotional strain has led to compromised decision-making and stress about survival; further supporting the current study’s findings [80]. Palinkas and Wong also found various health-related risk factors for developing or exacerbating mental illness amidst climate change, including sleep disruption, stating that 25% to 50% of individuals exposed to extreme weather events like extreme heat are more likely to experience adverse mental health outcomes and decreased work performance [81]. The literature also supports the finding that extended heat exposure has a significant impact on the health of outdoor workers, creating considerable stress. For instance, Martin et al. found that when heat exposure is combined with physical activity, individuals often experience cognitive impairment, where vigilance decreases, and more mistakes are common [82]. This indicates that heat-related cognitive decline may increase mental stress, and as such, highlighting the need for targeted interventions. The authors describe how physical discomfort from heat exposure limits the cognitive resources available to perform typically, likely causing irritability, reduced concentration and productivity. Lao et al. found that pressure from co-workers and superiors often caused reluctance in workers to adapt protective measures against the heat, wanting to maintain productivity and maintain their “masculinity” [83]. These findings extend the current study’s findings, suggesting pressure to maintain productivity and performance regardless of the weather conditions.

### Climate change and physical health

The intersection of climate change and physical health has become a critical area of concern, particularly for workers exposed to extreme heat [9,84]. Rising global temperatures have intensified occupational heat stress, leading to an increase in the prevalence of heat-related illnesses and injuries, many of which remain underreported [84–87].

According to findings in South Australia, the risk of occupational heat illness increases especially during heatwave conditions, with claims increasing four to seven times compared to non-heatwave periods [88]. Similar patterns were observed in agricultural settings, where Wagoner et al. found that extreme temperatures and inadequate hydration significantly contributed to heat strain and dehydration among workers who frequently face high workloads in hot conditions [52]. The prevalence of dehydration is alarming, with workers exhibiting urine-specific gravity levels representing clinical dehydration. For instance, Venugopal et al. discovered that high urine-specific gravity levels were found in 10.5% of the South Indian working population [57]. However, comparison across studies is limited by the variability in measurement methods and different study populations indicating heterogeneity. This aligns with the prevailing consensus in the literature, underscoring the need for targeted strategies to enhance hydration levels and manage heat stress effectively [89–91]. Potential interventions could include mandatory hydration breaks, and wearable heat sensors [92]. The impact of heat exposure extends beyond immediate effects and influences long-term injuries, such as chronic kidney disease (CKD). This finding is supported by broader research, which indicates that increased heat exposure can lead to harmful effects, particularly in tropical and subtropical regions [86,93,94].

These studies highlight the complexity of climate change on physical health, especially within worker populations. It is of the utmost importance to implement policies and strategies that support the prevention of heat-related illnesses and injuries [95,96]. It is essential to address health risks through effective interventions as heat-related events continue to grow and workers’ safety and health needs to be ensured [97].

### Climate change and work

In examining the relationship between climate change and work, our analysis revealed a strong relationship between climate change and loss of work, wages, GDP, labour capacity, and confidence in workplaces. The damaging impacts of heat exposure on physical health make it extremely difficult to maintain regular productivity levels; however, top-down pressure from supervisors or personal economic challenges make it challenging to fully implement protective measures. These restrictions inevitably result in losses in productivity and other factors. Parsons et al. found that heat exposure in regions that rely on deforestation is devastating, with nearly 4.9 million people losing more than 30 minutes of work each day; this is only expected to rise in the coming years [98]. Many studies have also found evidence of significant labour shortages due to climate change. For example, Hussain et al. found that some farmers in the Koshi River Basin in Nepal are facing a decrease in the amount of labour in the workforce at the most vital period for agriculture, also reporting nearly 6% of land entirely abandoned [99]. These reductions in labour capacity and working hours can significantly impact the local economy, resulting in several percent losses in Gross Domestic Product. Khan et al. found that Pakistan is expected to experience a loss of $ 19.5 billion by 2050 due to the underproduction of wheat and rice amid climate change, with similar global trends [100–103].

These impacts can be categorized into three main types of costs: direct employer costs, societal economic costs, and personal employee costs. Refer to Table 4 for a summarization of the three categories.

**Table 4:** Three cost categories and their descriptions.

Cost Category	Description
Direct Employer Costs	Sick pay, insurance premiums, overtime, and recruitment and replacement of absent or injured workers
Societal Economic costs	Lost productivity, lower outputs, reduced tax revenue and potential impact on national food security
Personal Employee Costs	Lost wage, barriers of career advancement opportunities and long-term health consequences.

The literature also supports the current study’s finding that supervisor behaviours put pressure on workers. Lingard et al. found that supervisors significantly influence workers’ health and safety and set the precedent for worker performance, creating norms and indirect expectations that workers may feel obligated to follow [104]. In addition, Kelley et al. found that many outdoor workers reported toxic behaviours from their employers, including wage theft, intimidation, and humiliation, which increased their risk of occupational injury and illness [105]. Many supervisors of outdoor workers seemingly prioritize productivity over the safety of their employees, potentially exacerbating labour shortages and reducing productivity. Research by Lim et al. also discussed how heat stress can cause heat-related illnesses and occupational injuries, which may lead to absenteeism and a loss of wages in the long ter [106]. These findings strongly support the current study’s results and suggest opportunities for workplace interventions, such as climate-specific scheduling, supervisor training that could protect worker health and maintain productivity [91].

### Limitations

This scoping review has several limitations to acknowledge when interpreting its findings. Our inclusion criteria limit us to studies written or translated in English, which may result in the omission of valuable data that could be offered in different languages. The ever-evolving nature of the research topic also raises a limitation, as there still might be knowledge gaps in the studies that were included. Therefore, future research should aim to incorporate a wider range of resources and tap into publications provided in other languages to offer a more comprehensive understanding of the impact of climate change on physical health, mental health, and productivity in various occupational contexts. More effective interventions can hereby be developed to address these challenges.

A further extension of that limitation is the review’s reliance on studies published in English. This introduces a potential geographic and linguistic bias. This may cause exclusion of important findings from non-English speaking regions that could provide unique insight into occupational health in the context of climate change. The findings presented here might not fully capture the global variation in workplace practices and strategies. Future research should consider multilingual searches and international collaborations with regional experts that can represent diverse geographic perspectives.

### Future research and implications

To mitigate the damaging impacts of climate change on occupational health, future studies should investigate the various factors that influence heat-related illnesses among workers. Effective interventions can hereby be implemented to protect employees from the harmful impacts of extreme heat. It is necessary to incorporate knowledge of specific resources, such as heat stress management strategies and cooling technologies, into future research to help workers cope with working under heat conditions.

As scientific knowledge of climate change continues to emerge, its significant impact on occupational health is becoming an increasingly important topic of study. While the current review focused on outdoor workers, future studies should expand beyond this population to include a broader range of occupational settings. This is critical as indoor and mixed-exposure workers can also face unique climate-related health risks that may be underrepresented in the literature. Longitudinal studies should be conducted to examine the continuing effects of climate change on mental and physical health. Longer-term projects (e.g., 5–10 years in duration) can provide a comprehensive review of how climate change impacts mental health, physical health, and productivity across various occupations. These studies can also include qualitative analysis on workers’ lived experiences of working under heat stress. These qualitative studies could shed light on the diverse challenges and coping mechanisms employed by workers, further enhancing our understanding of the implications of heat exposure on well-being and productivity. Emphasis should also be placed on functional outcomes, including occupational performance and the influence of mental and physical health on daily activities. There also needs to be a focus on functional outcomes, including work performance and the impact of mental and physical health on daily activities. Additionally, the definition of heat-related illness and its effects are not unified and agreed upon in the scientific community. We recommend that future research efforts aim to establish a consensus on terminology related to heat stress and its health outcomes. This clarification will improve communication among stakeholders and facilitate the design of targeted interventions.

Our findings highlight the need for government and workplace interventions to promote better care for workers’ physical and mental health in extreme heat conditions. Governments should establish and enforce minimum occupational standards for heat exposure, hydration, and breaks to support workplace adaptations. They should also provide evidence-based advisories for employers as a guide so that appropriate adaptations should be implemented. Furthermore, fiscal support must be issued for these adaptations and social security systems to ensure proper funding to accommodate these adaptations when necessary. Professional bodies should support these efforts by promoting ethical standards, certifying workplaces, and personnel to ensure best practice. Heat stress management should be prioritized and developed such that workers are allowed to work under humane conditions, including implementing workplace designs that improve ventilation and cooling.

Extrapolating from our findings, organizations may benefit from the implementation of monitoring systems to manage heat-related symptoms among outdoor workers. By doing so, workers can report their experiences with heat stress, including any mental or physical health issues, enabling earlier interventions. Proactively addressing these concerns can enhance worker productivity and well-being, while maintaining occupational efficiency. Research is also encouraged to support employers in implementing effective monitoring systems that align with best practices in occupational health and the workforce.

## Conclusion

This scoping review underscores the significant and multifaceted impacts of climate change on human health, particularly within occupational settings. The evidence presented indicates a concerning correlation between increasing temperatures and the rising incidence of heat-related illnesses among workers, particularly in high-risk environments, such as construction and agriculture. As temperatures continue to rise, the prevalence of heat stress and its associated health risks is expected to increase, highlighting the urgency for effective interventions. Workers also face heightened levels of anxiety and mental distress due to environmental changes and pressure to maintain productivity in extreme conditions. These factors compromise individual well-being and threaten overall workplace efficiency and safety. It is also essential to understand the unique vulnerabilities different worker populations face, particularly those in low-income and physically demanding roles. Specific strategies and solutions, including policies and organizational support, are essential for protecting these groups from the harmful effects of climate change.

It is crucial for stakeholders, including employers, health professionals, and policymakers, to instigate a plan of action that focuses on worker health and safety. By implementing proactive measures to manage heat stress and support mental well-being, organizations can foster healthier work environments that are more effectively equipped to address the challenges presented by climate change.

## Supporting information

S1 Checklist Preferred Reporting Items for Systematic reviews and Meta-Analyses extension for Scoping Reviews (PRISMA-ScR) Checklist. JBI = Joanna Briggs Institute; PRISMA-ScR = Preferred Reporting Items for Systematic reviews and Meta-Analyses extension for Scoping Reviews.
* Where sources of evidence (see second footnote) are compiled from, such as bibliographic databases, social media platforms, and Web sites.
† A more inclusive/heterogeneous term used to account for the different types of evidence or data sources (e.g., quantitative and/or qualitative research, expert opinion, and policy documents) that may be eligible in a scoping review as opposed to only studies. This is not to be confused with information sources (see first footnote).
‡ The frameworks by Arksey and O’Malley (6) and Levac and colleagues (7) and the JBI guidance (4, 5) refer to the process of data extraction in a scoping review as data charting.
§ The process of systematically examining research evidence to assess its validity, results, and relevance before using it to inform a decision. This term is used for items 12 and 19 instead of “risk of bias” (which is more applicable to systematic reviews of interventions) to include and acknowledge the various sources of evidence that may be used in a scoping review (e.g., quantitative and/or qualitative research, expert opinion, and policy document).
From: Tricco AC, Lillie E, Zarin W, O'Brien KK, Colquhoun H, Levac D, et al. PRISMA Extension for Scoping Reviews (PRISMAScR): Checklist and Explanation. Ann Intern Med. 2018;169:467–473. doi: 10.7326/M18-0850. This work is licensed under CC BY 4.0. To view a copy of this license, visit https://creativecommons.org/licenses/by/4.0/.
(PDF)

S1 Table Embase search strategy used to identify studies examining associations between climate-related exposures, occupational factors and health outcomes. / indicates controlled vocabulary terms (Emtree); .mp. indicates free-text searching across multi-purpose search fields; exp indicates exploded terms (includes narrower terms); Boolean operators (OR, AND) were used to combine search terms. Line numbers represent sequential search steps.
(PDF)

S2 Table CINAHL search strategy used to identify studies examining associations between climate-related exposures, occupational factors and health outcomes. MH indicates CINAHL subject headings; quotation marks indicate keyword search; S numbers indicate sequential search sets; Boolean operators (OR, AND) were used to combine terms.
(PDF)

S3 Table PsycINFO search strategy used to identify studies examining associations between climate-related exposures, occupational factors and health outcomes. / indicates controlled vocabulary subject headings; .mp. indicates free-text searching across multi-purpose search fields; exp indicates exploded terms (includes narrower terms); Boolean operators (OR, AND) were used to combine search terms. Line numbers represent sequential search steps.
(PDF)

S4 Table MEDLINE search strategy used to identify studies examining associations between climate-related exposures, occupational factors, and health outcomes. / indicates controlled vocabulary subject headings; .mp. indicates free-text searching across multi-purpose search fields; exp indicates exploded terms (includes narrower terms); Boolean operators (OR, AND) were used to combine search terms. Line numbers represent sequential search steps.
(PDF)

S5 Table Distribution of included articles across major themes. Each row represents an included study. Columns indicate the three major themes examined: (1) Climate Change and Mental Health; (2) Climate Change and Physical Health; (3) Climate Change and Work. An ‘X’ indicates that corresponding study addressed that theme.
(PDF)
