# Supplementary material for: The impacts of climate change on occupational health and work among outdoor workers: A scoping review
Source: PLOS Glob Public Health. 2026 Feb 6;6(2):e0005888. doi: 10.1371/journal.pgph.0005888 (PMC12880655; doi:10.1371/journal.pgph.0005888)
Supplement: S1 Table — / indicates controlled vocabulary terms (Emtree); .mp. indicates free-text searching across multi-purpose search fields; exp indicates exploded terms (includes narrower terms); Boolean operators (OR, AND) were used to combine search terms. Line numbers represent sequential search steps. (PDF) [file pgph.0005888.s002.pdf]

S1 Table. Complete Search Syntax for Embase

1	Job stress/
2	Mental stress/ or burnout/ or stress/
3	Job satisfaction/
4	Psychological stress.mp. or exp mental stress/
5	Mental health/ or psychological well-being/
6	Mental disease/di, pc, th [Diagnosis, Prevention, Therapy]
7	Mental illness.mp.
8	Health/ or global health/ or health status/ or men's health/ or mental health/ or minority health/ or population health/ or women's health/
9	Fatigue/ or chronic fatigue syndrome/ or exhaustion/ or lassitude/ or mental fatigue/ or overtraining syndrome/
10	Physical stress/ or physically induced stress/
11	Emotional stress/
12	Physiological stress/ or acute stress/ or chronic stress/ or physically induced stress/
13	1 or 2 or 3 or 4 or 5 or 6 or 7 or 8 or 9 or 10 or 11 or 12
14	Climate change/ or climate warming/
15	exp greenhouse effect/
16	Fossil fuel/
17	Ozone layer/
18	Global warming.mp.
19	exp air pollution/
20	exp traffic pollution/
21	exp radio frequency sputtering/ or radioactive air pollution/
22	Water pollution/
23	Climate emergency.mp.
24	Global climate change.mp.
25	exp carbon footprint/
26	exp greenhouse gas/
27	Exhaust gas/
28	Global heating.mp.
29	14 or 15 or 16 or 17 or 18 or 19 or 20 or 21 or 22 or 23 or 24 or 25 or 26 or 27 or 28
30	exp occupation/
31	Work engagement/ or social work/ or social work education/ or field work/ or work resumption/ or construction work/ or work experience/ or shift work/ or social work practice/ or social work student/ or work capacity/ or "work from home"/ or work-life balance/ or return to work/ or work disability/ or work schedule/ or work/
32	Job performance/
33	Workplace/
34	Work engagement/
35	30 or 31 or 32 or 33 or 34
36	13 and 29 and 35
