# Supplementary material for: The impacts of climate change on occupational health and work among outdoor workers: A scoping review
Source: PLOS Glob Public Health. 2026 Feb 6;6(2):e0005888. doi: 10.1371/journal.pgph.0005888 (PMC12880655; doi:10.1371/journal.pgph.0005888)
Supplement: S2 Table — MH indicates CINAHL subject headings; quotation marks indicate keyword search; S numbers indicate sequential search sets; Boolean operators (OR, AND) were used to combine terms. (PDF) [file pgph.0005888.s003.pdf]

**S2 Table. Complete Search Syntax for CINAHL**

| | |
|---|---|
| S1 | (MH “Stress, Occupational”) OR (MH “Stress, Physiological”) OR (MH “Minority, Stress”) OR (MH “Occupational Diseases”) OR (MH “Stress”) OR (MH “Occupational Health”) OR “occupational stress” OR (MH “Psychological Trauma”) OR (MH “Burnout, Professional”) |
| S2 | “job stress” OR (MH “Quality of Working Life”) OR (MH “Personal Satisfaction”) OR (MH “Mental Fatigue”) OR (MH “Burnout, Professional”) |
| S3 | “job fatigue” OR (MH “Fatigue”) |
| S4 | (MH “Mental Health”) OR “mental health” OR (MH “Mental Disorders, Chronic”) OR (MH “Organic Mental Disorders, Psychotic”) OR (MH “Mental Disorders”) OR (MH “Organic Mental Disorders”) OR (MH “Indigenous Health”) OR (MH “Behavioral and Mental Disorders”) OR (MH “Men’s Health”) OR (MH “Women’s Health”) OR (MH “Health”) OR (MH “Health and Disease”) OR (MH “Wellness”) OR (MH “Trauma”) OR (MH “Environmental Illness”) OR (MH “Disease”) |
| S5 | “mental illness” OR (MH “Acute Disease”) OR (MH “Critical Illness”) OR (MH “Chronic Disease”) OR (MH “Occupational Diseases”) OR (MH “Disease”) OR (MH “Undiagnosed Disease”) OR (MH “Wellness”) OR (MH “Syndrome”) OR (MH “Symptoms”) OR (MH “Health”) |
| S6 | “physical health” |
| S7 | “emotional exhaustion” OR (MH “Stress, Psychological”) OR (MH “Psychological Distress” OR (MH “Work Engagement”) OR (MH “Psychosocial Functioning”) OR (MH “Burnout, Professional”) OR (MH “Apathy”) |
| S8 | S1 OR S2 OR S3 OR S4 OR S5 OR S6 OR S7 |
| S9 | (MH “Climate Change”) OR (MH “Climate”) OR (MH “Rain”) OR (MH “Snow”) OR (MH “Greenhouse Effect”) OR (MH “Extreme Weather”) |
| S10 | (MH “Greenhouse Effect”) OR (MH “Sea Level Rise”) OR “global warming” |
| S11 | (MH “Environmental Pollution”) OR (MH “Air Pollution”) OR (MH “Radioactive Pollution”) OR (MH “Water Pollution”) OR (MH “Traffic Pollution”) OR (MH “Air Pollutants, Environmental”) OR (MH “Air Pollutants, Occupational”) OR (MH “Air Pollutants Radioactive”) OR (MH “Radioactive Pollutants”) OR (MH “Air Pollutants”) OR (MH “Persistent Organic Pollutants”) OR (MH “Environmental Pollutants”) OR (MH “Industrial Waste”) OR “pollution” OR (MH “Smog”) OR (MH “Ozone”) OR (MH “Radon”) |
| S12 | “climate emergency” |
| S13 | (MH “Natural Disasters”) OR (MH “Natural Environment”) OR (MH “Fossil Fuels”) OR (MH “Wildfires”) |
| S14 | “climate crisis” |
| S15 | (MH “Greenhouse Gases”) OR (MH “Carbon Footprint”) |
| S16 | “increased gas emissions” |
| S17 | “global healing” |
| S18 | S9 OR S10 OR S11 OR S12 OR S13 OR S14 OR S15 OR S16 OR S17 |
| S19 | “occupation” |
| S20 | (MH “Work”) OR “work” OR (MH “Work Environment”) OR (MH “Job Accommodation”) OR (MH “Work Engagement”) OR (MH “Job Re-Entry”) OR (MH “Job Satisfaction”) OR (MH “Shiftwork”) OR (MH “Job Experience”) OR (MH “Self Employment”) OR (MH “Women, Working”) OR (MH “Shift Workers”) |

| | |
|---|---|
| S21 | (MH “Job Performance”) OR “work performance” OR (MH “Work Environment”) OR (MH “Quality of Working Life”) OR (MH “Work”) OR (MH “Job Satisfaction”) |
| S22 | S19 OR S20 OR S21 |
| S23 | S8 AND S18 AND S22 |
