# Supplementary material for: The impacts of climate change on occupational health and work among outdoor workers: A scoping review
Source: PLOS Glob Public Health. 2026 Feb 6;6(2):e0005888. doi: 10.1371/journal.pgph.0005888 (PMC12880655; doi:10.1371/journal.pgph.0005888)
Supplement: S3 Table — / indicates controlled vocabulary subject headings; .mp. indicates free-text searching across multi-purpose search fields; exp indicates exploded terms (includes narrower terms); Boolean operators (OR, AND) were used to combine search terms. Line numbers represent sequential search steps. (PDF) [file pgph.0005888.s004.pdf]

S3 Table. Complete Search Syntax for PsycINFO

1	Occupational stress/ or burnout/ or stress/ or emotional exhaustion/ or employee well being/ or "quality of work life"/
2	Job stress.mp.
3	Stress/ or acute stress/ or chronic stress/ or physiological stress/ or psychological stress/ or trauma/ or anxiety/ or burnout/ or distress/ or "stress and trauma related disorders"/
4	Fatigue/
5	Mental health/ or health/ or emotional health/ or well being/
6	exp mental disorders/
7	Mental illness.mp.
8	exp physical health/
9	"quality of life"/ or "health related quality of life"/ or exp life satisfaction/
10	exp job performance/
11	Employee productivity/
12	Employee engagement/ or psychological engagement/ or employee efficiency/ or employee well being/ or job involvement/ or job satisfaction/
13	Job accommodation.mp.
14	1 or 2 or 3 or 4 or 5 or 6 or 7 or 8 or 9 or 10 or 11 or 12 or 13
15	Climate change/ or atmospheric conditions/ or climate anxiety/ or climate change attitudes/ or environmental measures/ or extreme weather/ or pollution/ or exp pro environmental behaviour/ or temperature effects/
16	Global warming/
17	Air pollution.mp.
18	Natural disasters/
19	Climate crisis.mp.
20	Greenhouse gas.mp.
21	Greenhouse effect.mp.
22	Global heating.mp.
23	15 or 16 or 17 or 18 or 19 or 20 or 21 or 22
24	exp occupations/ or occupational status/ or occupational success/
25	Working women/
26	Working men.mp.
27	Employed.mp.
28	Workplace.mp.
29	24 or 25 or 26 or 27 or 28
30	14 and 23 and 29
