# Supplementary material for: The impacts of climate change on occupational health and work among outdoor workers: A scoping review
Source: PLOS Glob Public Health. 2026 Feb 6;6(2):e0005888. doi: 10.1371/journal.pgph.0005888 (PMC12880655; doi:10.1371/journal.pgph.0005888)
Supplement: S4 Table — / indicates controlled vocabulary subject headings; .mp. indicates free-text searching across multi-purpose search fields; exp indicates exploded terms (includes narrower terms); Boolean operators (OR, AND) were used to combine search terms. Line numbers represent sequential search steps. (PDF) [file pgph.0005888.s005.pdf]

S4 Table. Complete Search Syntax for MEDLINE

1	Occupational stress/ or burnout, professional/
2	Stress, psychological/ or burnout, psychological/
3	Stress, Physiological/
4	Job stress.mp.
5	Workplace stress.mp.
6	Fatigue/ or mental fatigue/
7	Mental Health/
8	Mental Disorders/
9	Mental illness.mp.
10	exp Health/
11	Occupational Health/ or Women's Health/ or Men's Health/
12	Physical stress.mp.
13	Emotional exhaustion.mp.
14	"quality of life"/ or psychological well-being/
15	Psychological Distress/
16	Distress.mp.
17	Trauma.mp.
18	Work Performance/
19	Job performance.mp.
20	Work engagement/
21	Job Satisfaction/
22	Job accommodation.mp.
23	1 or 2 or 3 or 4 or 5 or 6 or 7 or 8 or 9 or 10 or 11 or 12 or 13 or 14 or 15 or 16 or 17 or 18 or 19 or 20 or 21 or 22
24	Climate change/ or global warming/
25	Water Pollutants, Chemical/ or Air Pollution/ or pollution.mp.
26	Climate emergency.mp.
27	Greenhouse Effect/ or global climate change.mp.
28	Climate crisis.mp.
29	Greenhouse Gases/
30	Increased gas emission.mp.
31	Global heating.mp.
32	Natural Disasters/
33	24 or 25 or 26 or 27 or 28 or 29 or 30 or 31 or 32
34	Occupations/
35	Workplace/ or working adult.mp. or Employment/
36	Work/
37	34 or 35 or 36
38	23 and 33 and 37
