# Supplementary material for: The impacts of climate change on occupational health and work among outdoor workers: A scoping review
Source: PLOS Glob Public Health. 2026 Feb 6;6(2):e0005888. doi: 10.1371/journal.pgph.0005888 (PMC12880655; doi:10.1371/journal.pgph.0005888)
Supplement: S5 Table — Each row represents an included study. Columns indicate the three major themes examined: (1) Climate Change and Mental Health; (2) Climate Change and Physical Health; (3) Climate Change and Work. An ‘X’ indicates that corresponding study addressed that theme. (PDF) [file pgph.0005888.s006.pdf]

S5 Table. Distribution of articles across major themes

Author, Year	Climate Change and Mental Health	Climate Change and Physical Health	Climate Change and Work
Al-Bouwarthan et al., 2019		X	
Al-Sayyad & Hamadeh, 2014		X	
Ayyappan et al., 2009	X	X	X
Berman et al., 2021	X		X
Brode et al., 2018		X	X
Crowe et al., 2010			X
Dally et al., 2020		X	X
Gellert et al., 2022	X	X	
Goodman et al., 2023		X	X
Han et al., 2021		X	X
Hansen et al., 2020	X	X	X
Hawkins & Ibrahim, 2023		X	X
Hunt et al., 2023			X
Ireland et al., 2023		X	
Kjellstrom et al., 2016			X
Kjellstrom et al., 2013		X	X
Krishnamurthy et al, 2017	X	X	X
Le Dang et al., 2014	X	X	X
Lee et al., 2018		X	X
Lin & Chan, 2009		X	X
Lohrey et al., 2021	X	X	X

Lundgren et al., 2014	X	X	X
Lundgren-Kownacki et al., 2018		X	X
Mansor et al., 2019	X	X	X
Marinaccio et al., 2019		X	X
Mathee et al., 2010	X	X	X
McInnes et al., 2017		X	
Meade et al., 2017	X	X	X
Moore et al., 2025	X		
Mutic et al., 2017		X	X
Nag et al., 2013		X	
Nunfam et al., 2020		X	
Nunfam et al., 2019		X	
Nyambe, 2024	X	X	X
Oyekale, A.S., 2015			X
Parker et al., 2024		X	X
Pires Biterncourt et al., 2020		X	
Pogacar et al., 2019		X	X
Polain et al., 2011		X	
Pradhan et al., 2019		X	
Pradhan et al., 2013		X	
Quiller et al., 2017		X	X
Rahimi et al., 2024		X	
Rahman et al., 2016		X	
Raval et al., 2018		X	X
Rudner et al., 2025		X	

Sahu et al., 2013		X	
Sett & Sahu, 2014		X	
Samaniego-Rascon et al, 2019		X	
Schifano et al., 2019		X	
Shanmugam et al., 2023		X	
Spencer et al., 2022		X	
Stoecklin-Marois et al., 2013		X	
Sverdlik et al., 2024		X	X
Tawatsupa et al., 2010	X	X	
Varghese et al., 2019		X	
Venugopal et al., 2021		X	
Venugopal et al., 2015		X	X
Wagoner et al., 2020		X	X
Xiang et al., 2014		X	
Xiang et al., 2016		X	
Xiang et al., 2015		X	
